# At the Crossroads: Mechanisms of Apoptosis and Autophagy in Cell Life and Death

**DOI:** 10.32607/actanaturae.11208

**Published:** 2021

**Authors:** V. L. Shliapina, S. V. Yurtaeva, M. P. Rubtsova, O. A. Dontsova

**Affiliations:** Shemyakin-Ovchinnikov Institute of Bioorganic Chemistry of the Russian Academy of Sciences, Moscow, 119997 Russia; NII Ajinomoto-Genetics, Moscow, 117545 Russia; Lomonosov Moscow State University, Moscow, 119991 Russia; Skolkovo Institute of Science and Technology, Moscow, 121205 Russia

**Keywords:** apoptosis, autophagy, telomerase, signaling pathways, regulation

## Abstract

Apoptosis and autophagy are conserved processes that regulate cell survival and death under stress conditions. Apoptosis aims to remove cells from the body with minimal damage to surrounding tissues. Autophagy promotes removal of damaged organelles, protein aggregates, and cellular pathogens, stimulating cell survival. The signaling pathways involved in the regulation of apoptosis and autophagy largely overlap, leading to both competition and unidirectional interaction, which is of particular interest in investigating them as potential targets for cancer, autoimmune, and neurodegenerative disease therapies. This review analyzes the main pathways of molecular interactions between autophagy and apoptosis, which is necessary for understanding the mechanism maintaining the balance between cell death and survival under unfavorable conditions.

## INTRODUCTION

Autophagy is a process that is stimulated by intracellular or environmental stress. The formation of autophagosomes and their fusion with lysosomes result in targeted degradation of damaged organelles, protein aggregates, and intracellular pathogens [1]. Investigating autophagy has become of great importance in the last decade, because the process is involved in the regulation of the metabolism of both the cell and the body. Dysregulation of autophagy affects the basic metabolic functions of cells, which can lead to the development of various diseases [2]. Now, there is reliable evidence that activation of autophagy by anticancer drugs can protect cancer cells from death, and that a decrease in the autophagy level is associated with the development of neurodegenerative and autoimmune diseases and general aging of the body [3].

Apoptosis is an evolutionarily conserved programmed mechanism of cell death, which selects cells during the normal development of eukaryotes and the maintenance of body homeostasis. Apoptosis is accompanied by morphological changes in the cell structure, which are associated with enzyme-dependent biochemical processes, as well as by the removal of cells from the body with minimal damage to the surrounding tissues [4].

Decreased cell apoptosis, coupled with a high proliferation level, can provoke the development of diseases such as cancer, while an accelerated rate of cell death promotes pathologies such as Alzheimer’s disease, Parkinson’s disease, and rheumatoid arthritis [4]. As we age, the efficiency of autophagy decreases [5, 6] while apoptosis increases in intensity [7]. Therefore, this review explores the molecular mechanisms regulating the cross-talk between apoptosis and autophagy.

## APOPTOSIS

**Figure 1:**
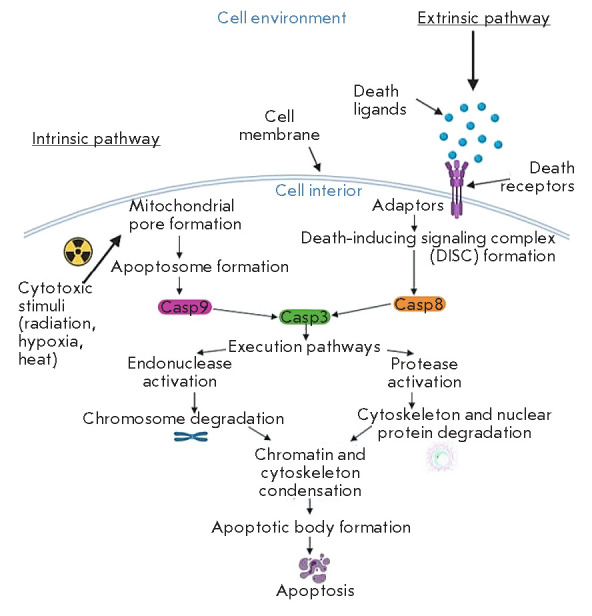
Scheme illustrating the intrinsic and extrinsic pathways of apoptosis activation (according to D’Arcy [4])

Apoptosis is a process of controlled death of the cell without spillage of its contents into the surrounding environment, which is called programmed cell death [4]. This process is regulated by proteins of the Bcl-2 family, which include both pro-apoptotic and anti-ap optotic components. The balance of these components determines cell life or death [8]. Stimulation of apoptosis leads to the activation of the pro-caspases that are the precursors of the cysteine-aspartic proteases known as caspases. There are two categories of caspases: initiator caspases and executioner caspases [9]. Specific signals indicative of cell damage stimulate the initiator caspases (caspases 8 and 9) that are activated by autoproteolysis and hydrolyze precursors of the executioner caspases (caspases 3, 6, and 7), ensuring that they remain active. Activation of the executioner caspases initiates a cascade of events that lead to the destruction of nuclear and cytoskeletal proteins, to protein crosslinking, the expression of ligands recognized by phagocytic cells, the formation of apoptotic bodies, and cell death [10, 11]. Apoptosis is accompanied by DNA fragmentation by endonucleases. The apoptosis process is highly conserved in multicellular organisms and is genetically controlled [12]. There are two pathways of apoptosis initiation: intrinsic and extrinsic. *Figure 1* shows the pathways of apoptosis.

The intrinsic pathway depends on factors released from mitochondria [13]; it includes various stimuli that act on several targets in the cell. The lack of cytokines, hormones, and growth factors leads to the activation of intracellular apoptosis activators from the Bcl-2 family, such as the p53 upregulated modulator of apoptosis (PUMA), Noxa, and BAX [4]. Under normal conditions, these proteins usually interact with anti-apoptotic proteins of the Bcl-2 family. In the absence of signals for survival and proliferation and upon exposure to hypoxia, toxins, radiation, reactive oxygen species, and viruses [14], the PUMA protein usually accumulates and its excess interacts with pro-apoptotic proteins of the Bcl-2 family, such as BAK and BAX. Their translocation into the mitochondrial membrane causes opening of the mitochondrial pore and relocation of pro-apoptotic proteins, such as cytochrome *c*, Smac/Diablo, and HtrA2/Omi, into the cytoplasm. Cytochrome *c*, a component of the mitochondrial respiratory chain, enters the cytoplasm, interacts with the apoptotic protease activating factor 1 (Apaf-1), and forms an apoptosome [15] that promotes the activation of initiator caspase 9, which triggers a cascade of apoptotic reactions. The mitochondrial proteins Smac/Diablo and HtrA2/Omi enter the cytoplasm and interact with inhibitors of apoptosis (IAP proteins), which promotes the release of caspases and their activation [4].

The extrinsic pathway of apoptosis activation is regulated by signaling cascades triggered by the death receptor (DR) [13]. Binding of death ligands, which are secreted by patrolling natural killer cells (NK cells) and macrophages or anchored on the surface of lymphocytes, to the DRs promotes interaction between the DR cytoplasmic death effector domain (DED) and monomeric procaspase 8 [16]. The resulting death-inducing signaling complex (DISC) provides proteolytic activation of caspase 8. The processed caspase induces the apoptosis-stimulating activity of endonucleases and proteases [4, 16].

The p53 transcription factor plays a key role in the regulation of apoptosis. p53 has a short lifetime, and its concentration in mammalian cells remains low through constant ubiquitination and subsequent degradation. But under stress conditions (DNA damage, hypoxia, cytokines, etc.), ubiquitination of p53 is inhibited and p53 is stabilized and accumulates in the nucleus. Various kinases provide for activated phosphorylation of p53. Depending on the conditions, these can be the kinases involved in cell cycle control (checkpoint kinases (Chk)) and cAMP-dependent protein kinase A (PKA), a regulator of lipid metabolism and glucose and glycogen levels; cyclin-dependent kinase 7 (CDK7) involved in cell cycle control and regulation of the transcriptional activity of RNA polymerase II; DNA-dependent protein kinase (DNA-PK), a mediator of the cellular response to DNA damage; and mitogen-activated protein kinases (MAPKs) such as Jun N-terminal kinase (JNK). Phosphorylation stimulates the oligomerization of p53, resulting in the formation of a tetramer. Tetrameric p53 activates the expression of genes whose promoter regions contain sites for interaction with p53 [17, 18]; e.g., Fas ligand genes [19, 20] and the *DR5 *gene encoding a death receptor interacting with tumor necrosis factor family cytokines TRAIL (TNF-associated apoptosis-inducing ligand). Involvement of p53 in the intrinsic pathway of apoptosis is associated with the Bcl-2 family proteins that regulate the release of cytochrome* c *from mitochondria. The key pro-apoptotic Bcl-2 genes include *BAX*, *Noxa*, *PUMA*, and *BID*, which are targets for p53 [21].

## AUTOPHAGY

During autophagy, various cellular components or even entire organelles enter lysosomes that contain enzymes that hydrolyze engulfed components [4]. Autophagy is stimulated in response to various factors such as ATP and nutrient deficiency or signals originating on the surface of damaged organelles or regulating cell differentiation during embryogenesis [22]. The autophagy process underlies adaptive and innate immunity. For example, destruction of intracellular pathogens, delivery of antigens to MHC class II holding compartments, and transport of viral nucleic acids to Toll-like receptors involve autophagosomes [23]. Although autophagy is often used to recycle cellular components, it can also lead to cell destruction. Therefore, autophagy is associated with the removal of senescent cells from tissues and the destruction of tumor lesions [22]. A low efficiency of autophagy is associated with the development of cancers and, especially in old age, the accumulation of protein aggregates in neurons and the development of neurodegenerative conditions, including Alzheimer’s [24]. Autophagy activation in rapidly proliferating cells facilitates the overcoming of deficiency in the intracellular components necessary for biosynthesis [25]. An increased autophagy level, often present in cancer cells, enables the cells to function more efficiently under nutrient deficiency and also reduces their sensitivity to cytotoxic substances [26].

**Figure 2:**
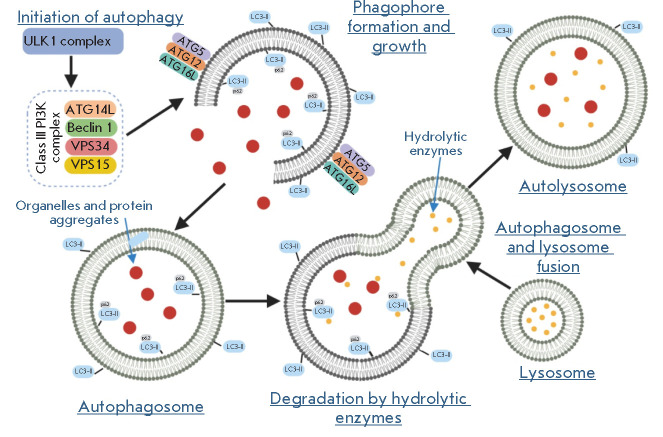
General scheme of autophagolysosome formation (according to D’Arcy [4]). Activation of ULK1 and class III PI3K complexes stimulates autophagophore formation. A complex consisting of ATG5, ATG12, and ATG16L stimulates, together with LC3II, phagophore lengthening and is necessary for autophagosome formation. The p62 protein associates with LC3II and ubiquitinated degraded proteins and is engulfed by the autophagosome. Lysosomal enzymes hydrolyze the autophagosome contents after fusion

There are three different forms of autophagy: macroautophagy, microautophagy, and selective autophagy. In macroautophagy, whole regions of the cell are enclosed in double-membrane vesicles called autophagosomes. Autophagosomes fuse with lysosomes to form autophagolysosomes, the contents of which are degraded by hydrolytic enzymes [27]. *Figure 2* presents a general schematic of autolysosome formation [4].

**Pathways of autophagy regulation**

At the first stages of autophagy, the ULK1 complex consisting of Unc-51-like autophagy-activating kinase 1 (ULK1), autophagy-related protein 13 (ATG13), focal adhesion kinase family interacting protein of 200 kDa (FIP200), and ATG101 is translocated to the autophagy initiation sites and regulates the recruitment of the VPS34 (vacuolar protein sorting 34) complex. VPS34, composed of class III phosphoinositide 3-kinase (PI3K), VPS34, ATG14L, VPS15, and Beclin 1 (*Fig. 2*), provides for the formation of phosphatidylinositol-3-phosphate (PI3P) at phagophore formation sites. PI3P initiates the binding of a number of proteins that form the autophagosome. The formed phagophores gradually increase through two ubiquitin-like conjugation cascades: ATG5-ATG12 and MAP-LC3/ATG8/LC3. The phagophore, as it elongates, engulfs part of the cytoplasm, forming a double-membrane autophagosome by self-closure. Finally, fusion of the autophagosome with the lysosome leads to the formation of an autolysosome and degradation of the contents, and the produced macromolecular blocks are released into the cytosol and can be re-used by the cell as building blocks [28]. The central regulator of autophagy is the mammalian target of rapamycin (mTOR) kinase. Suppression of mTOR activity stimulates ULK1 complex formation and activates autophagy.

Activation of autophagy by internal or external stimuli is subject to multistep regulation that involves the main cellular signaling cascades. The most studied modulators of autophagy are the PI3K, AKT, and AMPK kinases that regulate cell proliferation, metabolism, and survival. Activation of the PI3K/AKT/ mTOR-mediated signaling pathway usually inhibits autophagy [29]. This signaling cascade is modulated by the phosphatase and tensin homolog (PTEN), insulin, Sirt1, 5’-AMP-activated protein kinase (AMPK), p38 mitogen-activated protein kinase (p38 MAPK), and p53. Stimulation of autophagy is facilitated by the activation of the MAPK signaling pathway Ras/Raf/ERK that regulates the activity of the JNK kinases involved in the modulation of proliferation, differentiation, inflammation, and apoptosis. Activating mutations in the *Ras *or *B-Raf *oncogenes are often associated with a malignant transformation of cells, and JNKs regulate apoptosis through post-translational phosphorylation of Bcl-2 [30, 31].

**Figure 3:**
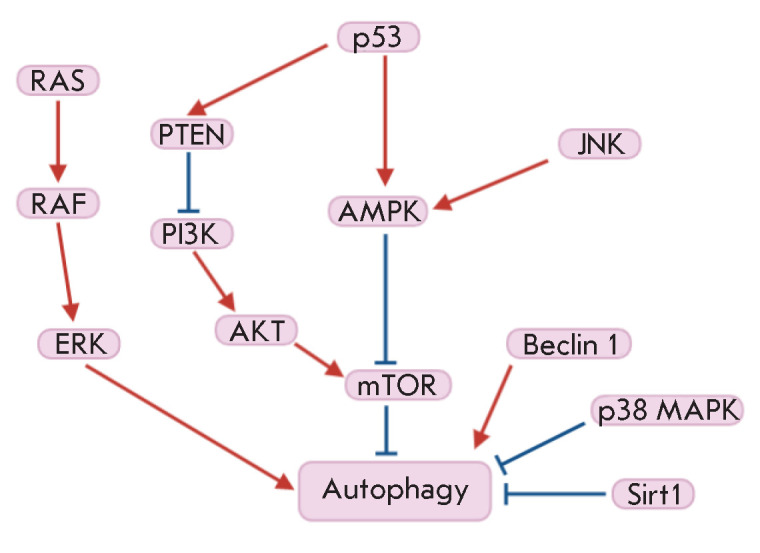
Scheme illustrating the regulatory mechanisms of autophagy (according to Jiang [1])

*Figure 3* displays the mechanisms regulating autophagy.

## AUTOPHAGY AND APOPTOSIS: MOLECULAR INTERACTIONS

Both autophagy and apoptosis play an important role in the development processes, maintenance of tissue homeostasis, and the pathogenesis of many diseases. To date, there is growing evidence that the main molecular components of the signaling pathways of autophagy and apoptosis are in complex cross-talk and are often induced by similar stimuli. For example, experiments have demonstrated that both apoptosis and autophagy are activated in response to metabolic stress [32] or exposure to reactive oxygen species [33]. Interesting data on the cross-talk between autophagy and apoptosis were obtained by the analysis of the molecular mechanisms of endoplasmic reticulum stress. The adaptive response of cells to the disruption of calcium homeostasis or endoplasmic reticulum dysfunctions is an enhancement of autophagy and apoptotic cell death [34].

There are several key variants of functional interactions between apoptosis and autophagy. In the case of “partnership relationships,” apoptosis and autophagy act in the same way, leading to cell death. In the case of an “antagonistic relationship,” apoptosis and autophagy are processes with different goals. In this case, autophagy does not lead to cell death and, moreover, reduces the efficiency of apoptosis, providing conditions favorable to cell survival. In the case of “activating Relationships”, autophagy promotes the apoptotic program, ensuring certain stages, without leading to cell death in itself [35].

Therefore, autophagy and apoptosis can interact, counteract, or facilitate each other, affecting the cell’s fate in different ways. In this case, there are several main molecular pathways that provide for complex functional interactions between autophagy and apoptosis.

**Beclin 1 regulates the choice between autophagy and apoptosis**

**Figure 4:**
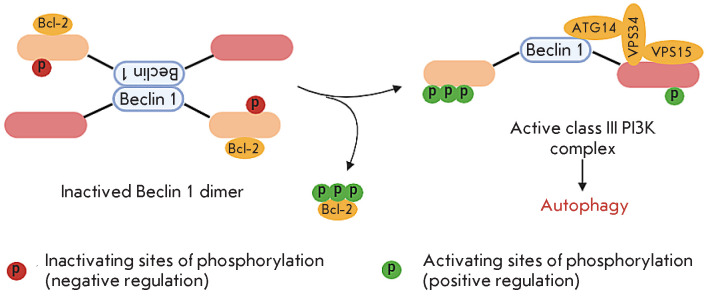
Scheme illustrating the conversion of an inactive Bcl-2–Beclin1 complex into the active PI3K complex (according to Menon [41])

An important component of the pre-autophagosome is the Beclin 1 protein that plays a regulatory role in choosing the stress response mechanism. The Bcl-2 protein family (Bcl-2, Bcl-xL, and Mcl-1) includes well-known anti-apoptotic mediators whose role in suppressing autophagy is under study. The cytoprotective function of Bcl-2 proteins is related to their ability to interact with BAX and BAK and, thus, prevent apoptosis [36]. The Beclin 1 protein contains a BH3 domain homologous to the Bcl-2 domains. This protein determines the fate of cells under stress by modulating the interaction of autophagy and apoptosis. Beclin 1 recruits key autophagic proteins into the pre-autophagosomal structure [4]. The Beclin 1 BH3 domain is responsible for interaction with anti-apoptotic members of the Bcl-2 family (*Fig. 4*), which interferes with the assembly of the pre-autophagosomal structure and leads to the inhibition of autophagy [36]. Under starvation stress, JNK kinase phosphorylates Bcl-2, which promotes dissociation of the Bcl-2–Beclin 1 complex, followed by pre-autophagosomal structure assembly and autophagy [37]. Prolonged activation of the JNK cascade and Bcl-2 phosphorylation lead to apoptosis, due to caspase 3 activation [36]. Kinases, such as the cell-death-associated protein kinase (DAPK), Rho-associated kinase 1 (ROCK1) involved in the regulation of cell proliferation, inflammation, and adhesion [38, 39], as well as MK2 and MK3, which serve as substrates for p38 MAPK [40], were shown to perform inhibitory phosphorylation of the Beclin 1 BH3 domain and block the assembly of the pre-autophagosome. The stimulating effect is exerted by kinase Mst1, a regulator of effector T cell activity and regulatory T cell differentiation. Phosphorylation of the Beclin 1 BH3 domain by Mst1 kinase promotes the Bcl-2–Beclin 1 interaction [38], thereby preventing the assembly of the class III PI3K complex, which leads to the inhibition of autophagy. *Figure 4* presents a schematic of activated class III PI3K complex formation.

**mTOR kinase signaling pathways**

One of the points of molecular interaction between the autophagy and apoptosis pathways is mTOR kinase, a serine/threonine kinase from the phosphatidylinositol kinase family, which plays an important role in the regulation of growth and aging processes. The mTOR kinase activity changes depending on external and internal factors: presence/absence of nutrients, ATP, growth factors, and stress factors [1].

mTOR is known to be involved in two complexes: mTORC1 consisting of mTOR, mLST8, DEPTOR, RAPTOR, and PRAS40; mTORC2 consisting of mTOR, mLST8, DEPTOR, RICTOR, mSIN1, and PROTOR. mTORC1 phosphorylates ribosomal protein S6 kinase (p70 S6K1) and translation initiation factor 4E-binding protein 1 (4EBP1) and stimulates protein biosynthesis. mTORC1 performs regulatory phosphorylation of ULK1, which inhibits autophagy, and is involved in lipid metabolism via a modification of Lipin 1 phosphatidate phosphatase. The mTORC2 complex was discovered relatively recently. mTORC2 is activated in response to growth factors, and its substrates are AKT kinase, serum and glucocorticoid-inducible kinases (SGKs), and protein kinase C (PKC), a component of the regulatory cascade activated by G-protein-coupled growth factor receptors [42].

mTOR activity is regulated by the small GTP-binding protein Rheb. After binding to GTP, Rheb activates mTOR. GTP hydrolysis stimulated by the TSC1/TSC2 (tuberous sclerosis) complex leads to the inactivation of Rheb and mTOR, respectively. Inhibition of autophagy by the regulatory phosphorylation of TSC1/TSC2 is mediated by various factors. For example, AKT and MAP kinases, extracellular signal-regulated kinases (ERK), and p90 ribosomal S6 kinase (RSK), which perform inactivating phosphorylation of Ser939 TSC2, inhibit autophagy; AMPK, which phosphorylates Ser1387 TSC2, stimulates autophagy [35].

Stress or nutrient and energy deficiency in the cell leads to the inhibition of mTOR activity and, therefore, to the induction of autophagy [1]. However, prolonged starvation leads to mTOR reactivation and, consequently, to the inhibition of autophagy [43].

addition, mTOR has a pleiotropic effect on apoptosis, in particular through the p53, BAD, and Bcl-2 proteins [44]. The interaction between Bcl-2 and Beclin 1 inhibits autophagy and prevents the regulation of the expression of pro-apoptotic protein genes by the p53 protein [45]. MCL1, one of the Bcl-2 family proteins, has been shown to act as a stress sensor that simultaneously controls both autophagy and apoptosis in neurons [46, 47].

**Figure 5:**
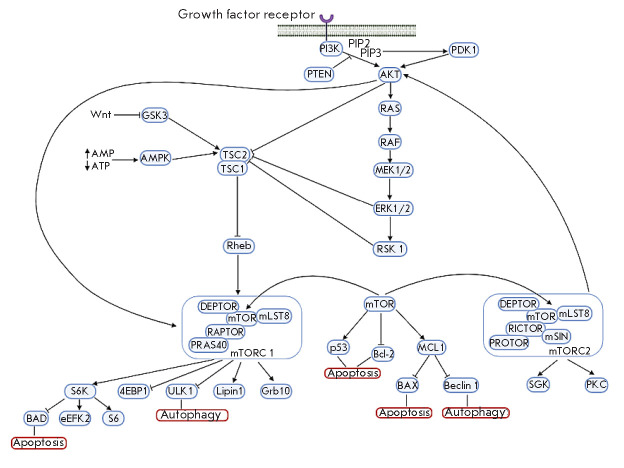
Scheme illustrating the mTORregulated pathways of autophagy and apoptosis (according to [35, 42, 48, 49])

*Figure 5* shows the pathways of interaction between autophagy and apoptosis with the involvement of mTOR.

**p38 MAPK signaling pathway**

p38 MAPK plays an important role in the regulation of apoptosis, cell cycle, and growth and differentiation processes and serves as a target for a number of drugs (e.g., cyclophosphamide, oxaliplatin). But under certain conditions, p38 MAPK can also mediate resistance to apoptosis (through activation of COX-2, etc.) [50]. p38 MAPK-regulated signaling pathways are activated in response to a wide range of stimuli, such as mitogenic factors (e.g., growth factors or cytokines), environmental signals, and genotoxic stress. After exposure to these stimuli, p38 MAPK is activated by the upstream kinases MKK3 and MKK6. Sometimes, p38 can also be phosphorylated by MKK4 kinase, which is well known as a JNK activator [51].

In addition to apoptosis, p38 MAPK is involved in the regulation of autophagy in response to chemotherapeutic agents [52]. The molecular mechanisms of the interaction between p38 and autophagy remain largely unknown. By phosphorylating Atg5, p38 MAPK is known to be able to inhibit the autophagy caused by a lack of nutrients [53]. Also, p38 MAPK can negatively regulate macroautophagy during cell growth in a normal medium containing amino acids and serum (basal autophagy) [54], and autophagy caused by nutritional deficiencies [55, 56]. Activation of p38 MAPK signaling induces autophagy to maintain cell survival through phosphorylation of GSK3β kinase from the serine/threonine kinase family, which is involved in the regulation of energy metabolism [57].

p38 MAPK is believed to be the main factor involved in maintaining a balance between p53-dependent apoptosis and autophagy under genotoxic stress induced by 5-fluorouracil [58].

However, there are contradictory data on the potential role of p38 MAPK in autophagy and apoptosis processes. Reactive oxygen species can induce oxidative stress that enhances autophagy and decreases apoptosis [59]. MAPK was found to play a vital role in the transition from autophagy to apoptosis in human colon cancer cells treated with MS-275, a histone deacetylase inhibitor. A high level of *p38 *expression is associated with the activation of autophagy, while low expression of this gene induces apoptosis. Therefore, the p38 MAPK signaling pathway can play a critical role in choosing one of the two cellular processes triggered by chemotherapy-induced genotoxic stress [50].

**JNK signaling pathway**

JNK kinase, also known as a stress-activated protein kinase (SAPK) of the MAPK family, is initially activated in response to various stress signals and is involved in many cellular processes, including apoptosis and autophagy. Under genotoxic stress conditions, JNK is a positive regulator of both apoptosis and autophagy [50].

JNK regulates apoptosis through two different mechanisms. On the one hand, it promotes phosphorylation of c-Jun and the transcription factor ATF2, which activates the transcription factor AP-1 (activator protein 1) and expression of the genes associated with the signaling pathway regulated by Fas death receptors. Binding of the FasL ligand to the Fas receptor can mediate the activation of caspase 8 that processes effector caspase 3, initiating apoptosis. On the other hand, JNK provides phosphorylation of Bcl-2/Bcl-xL anti-apoptotic proteins, which changes the mitochondrial membrane potential and promotes the release of cytochrome *c*, activation of caspases 9 and 3, and induction of apoptosis [60].

Bcl-2/Bcl-xL phosphorylation stimulates autophagy through the dissociation of the Beclin 1–Bcl-2/Bcl-xL complex [61]. On the other hand, JNK activates the damage-regulated autophagy modulator (DRAM). DRAM is a target of p53, and DRAM induction under genotoxic stress conditions [62] stimulates autophagy by blocking the fusion of autophagosomes with DRAMcontaining lysosomes [63, 64].

In general, the results of the studies carried out to date indicate a significant overlap or mutual dependence of the intracellular signaling mechanisms involved in the regulation of JNK-mediated apoptosis and autophagy. However, the question of how JNK controls the balance between apoptosis and autophagy in response to genotoxic and oxidative stress remains open [50].

**Figure 6:**
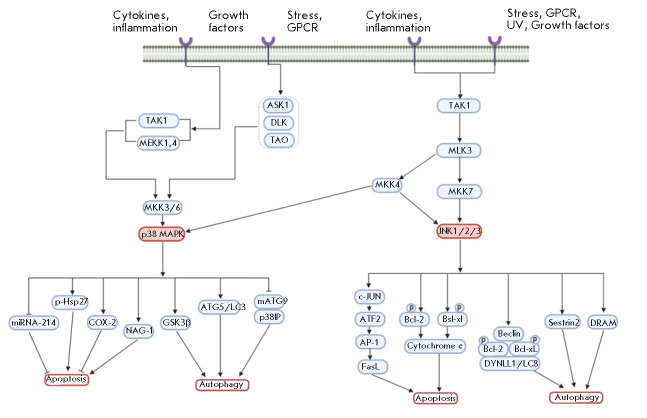
Scheme of p38 MAPK and JNK signaling pathways, which illustrates their role in the regulation of autophagy and apoptosis (according to [50])

*Figure 6* illustrates the role of JNK and p38 MAPK signaling pathways in the regulation of autophagy and apoptosis.

## AUTOPHAGY AND APOPTOSIS IN AGING

Aging of an organism is a complex process involving a disruption of and decrease in the functions of many systems both at the whole organism level and at the cellular level [65]. All these processes ultimately lead to the death of the body and the development of many diseases, including the metabolic syndrome, neurodegenerative diseases, and cancer [6]. Aging of cells is accompanied by shortening of telomeres [66, 67], a decrease in the efficiency of autophagy [5, 6], and excessive activation of apoptosis [7]; however, the mechanisms of these processes remain not fully understood.

Telomeres can be lengthened by a specialized complex, telomerase. The complex includes reverse transcriptase (TERT), RNA telomerase (TERC), and additional proteins involved in the assembly of the enzyme and regulating its activity. The complex is active in cells characterized by a high proliferation rate, such as bone marrow cells, activated lymphocytes, gametes, and cancer cells, while telomerase is inactive in most somatic cells [66]. Expression of the *TERC *gene in cells lacking telomerase activity suggests that RNA telomerase performs some additional functions unrelated to telomerase activity and telomere elongation. Under stress conditions, TERT shuttles from the nucleus into mitochondria and promotes the protection of cells [68]. Increased expression of the telomerase component genes stimulates expression of the hexokinase 2 gene and activates autophagy through mTOR inhibition [69, 70]. Deletion of the *mTERC *gene in mice leads to mTOR activation and a constantly increased level of S6K1 phosphorylation. Inhibition of mTORC1 by rapamycin reduces the lifespan of these mice, but not that of wildtype mice [71].

Transcription of the human telomerase RNA gene produces an elongated precursor [72] that contains an open reading frame encoding the hTERP protein [73]. An increased level of the hTERP protein protects cells under conditions of apoptosis induction, while hTERP mutations affect processing of the LC3 protein, one of the main participants in autophagosome formation [72, 73, 74]. hTERP is involved in the regulation of molecular interactions between autophagy and apoptosis, as well as in the adaptation of cells to stress conditions [73]. The molecular mechanisms of the influence of telomerase components on autophagy are under active study.

## CONCLUSION

To conclude, it is clear that autophagy and apoptosis are in a complex functional relationship that varies from cooperation to antagonism in different tissues and under different conditions. The balance between autophagy and apoptosis is maintained by a complex system of interactions between many signaling pathways, which involve both key proteins of autophagy and apoptosis (Beclin 1, caspase, p53, etc.) and polyfunctional regulatory molecules (e.g., mTOR, p38 MARK, or JNK). But it should be noted that clinical and experimental data on the autophagy and apoptosis ratio in normal tissues and various pathological conditions, including malignant tumors, are for the most part contradictory, and that the topic of balance between apoptosis and autophagy needs further investigation.
